# Exploring the experience of being viewed as “not sick enough”: a qualitative study of women recovered from anorexia nervosa or atypical anorexia nervosa

**DOI:** 10.1186/s40337-021-00495-5

**Published:** 2021-10-30

**Authors:** Kari Eiring, Trine Wiig Hage, Deborah Lynn Reas

**Affiliations:** 1 Institute of Psychology, Faculty of Social Sciences, University of Oslo, Oslo, Norway; 2 Regional Department of Eating Disorders, Division of Mental Health and Addiction, Oslo University Hospital-Ullevål, P.O. Box 4956, 0424 Nydalen Oslo, Norway

**Keywords:** Eating disorders, Anorexia nervosa, Atypical anorexia nervosa, The thin ideal, Not sick enough

## Abstract

**Background:**

Despite common misconceptions, an individual may be seriously ill with a restrictive eating disorder without an outwardly recognizable physical sign of the illness. The aim of this qualitative study was to investigate the perspectives of individuals who have previously battled a restrictive eating disorder who were considered “not sick enough” by others (e.g., peers, families, healthcare professionals) at some point during their illness, and to understand the perceived impact on the illness and recovery. Such misconceptions are potentially damaging, and have been previously linked with delayed help-seeking and poorer clinical outcomes.

**Methods:**

Seven women who had recovered from anorexia nervosa or atypical anorexia nervosa participated in semi-structured interviews. Interviews were transcribed and interpretive phenomenological analysis was used.

**Results:**

Three main themes emerged: (1) dealing with the focus upon one’s physical appearance while battling a mental illness, (2) “project perfect”: feeling pressure to prove oneself, and (3) the importance of being seen and understood. Participants reported that their symptoms were occasionally met with trivialization or disbelief, leading to shame, confusion, despair, and for some, deterioration in eating disorder symptoms which drove further weight loss. In contrast, social support and being understood were viewed as essential for recovery.

**Conclusion:**

To facilitate treatment seeking and engagement, and to optimize chances of recovery, greater awareness of diverse, non-stereotypical presentations of restrictive eating disorders is needed which challenge the myth that weight is the sole indicator of the presence or severity of illness.

## Plain English Summary

A persistent myth is that restrictive eating disorders are outwardly recognizable due to severely low body weight or emaciation. Atypical anorexia nervosa (AAN) and anorexia nervosa (AN) are both characterized by restrictive eating behavior. Individuals with AAN do not have current low body weight, although some research suggests they have higher levels of impairment and eating disorder pathology than their peers with AN, and their physical health may be equally compromised. Despite this, individuals with AAN are more likely to have a longer duration of illness and less likely to receive inpatient care, suggesting their illness is not always recognized by others. Additionally, thinness is highly valued in today’s society and restrictive eating behavior or “dieting” is commonplace, which may promote trivialization, or even reinforcement, of initial weight loss by friends or family. With this in mind, our study aimed to explore the experiences of seven recovered individuals recovered from AN or AAN who were directly or indirectly told by peers, families, or healthcare professionals that they were “not sick enough” at some point during their course of illness. We explored the perceived effects on symptoms, motivation for treatment, mental health and well-being. Participants reported their symptoms were occasionally trivialized or dismissed, leading to shame, confusion, and self-doubt regarding the seriousness of their symptoms. For some, a deterioration in eating disorder symptoms ensued that led to additional weight loss, sometimes in a competitive or perfectionist pursuit to “succeed” at eating restrictively. Findings demonstrated the value of being seen and understood, as well as the potential damage of being considered as “not sick enough” by others when battling a restrictive eating disorder.

## Introduction

A prevailing misconception is that an eating disorder, particularly those marked by restrictive eating, is always outwardly recognizable due to malnutrition or emaciation [1]. In reality, restrictive eating disorders occur across a range of body weights [2, 3]. For instance, Atypical Anorexia Nervosa (AAN), currently categorized as an Other Specified Feeding and Eating Disorder in the DSM-5 (OSFED) [4] and formerly categorized under ED Not Otherwise Specified in the DSM-IV (EDNOS) [5], shares the same criteria as AN, but individuals are within or above normal body mass index range (BMI) despite undergoing significant weight loss. This group is diverse, inclusive of boys and men, persons who are overweight or obese prior to illness [6], or those in a prodromal or residual phase of AN. Despite normal weight status, individuals with atypical AN are at risk for medical instability and malnourishment, with a similar array of medical comorbidities similar to AN, including low bone mineral density, bradycardia and hypotension [7–10]. Moreover, some evidence suggests that individuals with atypical AN have even higher levels of eating disorder psychopathology than AN, including greater distress related to body image concerns [6].

Delays in treatment attributable to trivialization of symptoms by healthcare professionals, or stringent weight-based criteria to access treatment, have been previously identified as barriers to early intervention [11, 12]. Early detection and early intervention are key to making a full recovery [13, 14], yet individuals with atypical presentations often endure a longer duration of illness [15–17] and are less likely to receive inpatient care [18], suggesting underrecognition. Furthermore, sociocultural environments in which thinness is highly valued, restrictive eating behavior or “dieting” is normative [19] and weight-based stigma is pervasive [20] may promote trivialization, or even reinforcement, of initial weight loss by peers, family, and healthcare providers.

Eating disorders are typically furtive illnesses, and treatment seeking overall is strikingly low, with as few as 17–31% of persons in the community with a diagnosable eating disorder seeking treatment [21]. While the number of individuals with atypical presentations seeking treatment is increasing, i.e., up five-fold in five years at one acute inpatient adolescent setting [3], a recent systematic review found that AAN remains underrepresented in clinical samples compared to epidemiological samples [22]. One study investigating treatment-seeking characteristics amongst a sample of US adolescents estimated that only 20% of their sample sought treatment for their illness, and individuals with “counter-stereotypical” symptoms were the least likely to seek treatment [23]. Although the vast majority of clinical studies have included predominantly white female adolescents with limited diversity [22], atypical AN presentations are also not uncommon among adults in diverse population samples [24], such as military Veterans [25], yet active help-seeking is similarly low. Anecdotal evidence from clinical observations and personal accounts in “Almost Anorexic” by Thomas and Schaefer [26] suggest that individuals struggling from eating problems may forego seeking help due to fears of not being “sick” or “thin” enough to deserve or warrant treatment. While some individuals may indeed present with less severity or impairment than full-criteria AN resulting in less frequent referral or admission, those who are actively seeking treatment are likely experiencing a significant level of distress or concern over their eating difficulties, and would benefit from clinical attention.

Little is known regarding the experiences of individuals who have actively sought help for a restrictive eating disorder and the perceived impact of delayed, denied, or diminished treatment opportunities due to others perceiving them as “not sick or thin enough.” Some research has found that former patients had experienced referral and admission criteria to be exceedingly weight-based, setting “a threshold that appeared to *promote* weight loss as a means of accessing support…” [27]. Similarly, a qualitative study undertaken by the Academy of Eating Disorders reported that some service users perceived the necessity of being “physically on death’s door” in order to gain access to treatment [28], and this may remain a salient issue across all phases of the illness and recovery. Relying upon physical measures of illness to determine discharge readiness, for instance, may result in relapse if not accompanied by improvements in other domains. Additionally, waiting until significant weight loss occurs prior to resumption of treatment readmission may undermine patient motivation, leaving individuals unsupported in their efforts to manage a relapse.

The aim for this qualitative study was to understand the perspectives of individuals with a past history of diagnosed AN or AAN, who were either directly or indirectly told by others they were “not sick enough” at some point during the course of their illness. Individuals with either diagnosis were included due to diagnostic fluctuation or progression which may occur between AAN and AN over time [29], and to allow for possible inconsistency in diagnostic practices between providers, as AAN and AN diagnostic criteria are not always uniformly applied in clinical settings [30]. Qualitative research is particularly well-suited to gather in-depth insights from persons with lived experiences to concretize important directions for future research and to inform clinical practice [31, 32], To date, no study has specifically investigated the experience, and perceived impact, of being viewed by others as “not sick enough” with an ED. This is despite indications that some individuals delay help-seeking due to worries about the legitimacy of their eating problems, or some may deliberately—and dangerously—intensify efforts to lose weight to “prove” their ED or access treatment [28]. Understanding the views of persons with lived experiences informs hypotheses and outlines priorities for future studies, and may guide policy that reduces the overall suffering attributable to eating disorders by facilitating earlier or appropriate intervention.

## Method

### Sample

The participants were non-randomly selected using purposive, non-probability sampling via online adverts on social media (Facebook, Twitter) of Norway’s two largest non-profit ED organizations (SPISFO, ROS) and the Regional Department of Eating Disorders (RASP) at Oslo University Hospital, which offers tertiary, specialized ED treatment to a catchment area of approximately 2.9 million people. Individuals aged 18 years and older who had previously received a diagnosis of AN or AAN and had at least one prior experience of being perceived as “not sick enough” by others (e.g., family, friends, peers, teachers, healthcare professionals) were eligible for inclusion. Individuals who self-reported a *current *ED diagnosis were not eligible for inclusion. Being perceived as “not sick enough” was not formally operationalized, but based upon the participant’s self-reported subjective experiences. Participants were instructed to reflect only upon experiences in which *others* perceived them as not sick enough, not the times when they personally didn’t consider themselves ill, as denial or lack of recognition of the seriousness of the illness is diagnostic [4]. Respondents to the study advertisement were contacted by email and screened by phone. Recruitment was terminated with theoretical saturation [33, 34]. Participation was voluntary, with no financial compensation. Seven females participated, ranging age from 21 to 47 years, with a mean age of 28 years and median 25 years. Written consent was obtained from all participants. The study was conducted in accordance with the Helsinki convention and ethical approval was obtained from the Regional Ethics Committee (REK) and the Internal Review Board at Oslo University Hospital.

### Procedure

Semi-structured interviews for this study were conducted in-person or over the phone (n = 2) on a one-to-one basis by KE. Qualitative interviews were considered well-suited to address the research aim by allowing the interviewer to gain insight into the subjective experience of the participant and provides a comprehensive set of data [35]. Care was taken to establish rapport and promote open discussion. Interviews were semi-structured around a topic guide that covered two main points: narration of the journey with an ED and experiences and reflections specific to the research question (e.g., “Can you describe your experience(s) of being viewed by others as “not sick enough”? I wondered if you could describe any specific instances when this occurred. Again, we’re interested in your experiences when *others* perceived you as “not sick enough”––not the times when you *personally* didn’t consider yourself ill with an eating disorder.”). The topic guide (available upon request) was iteratively developed based on feedback from user representatives from SPISFO, ROS, and treatment providers at RASP, and was piloted with a volunteer who had lived experience with an ED. The average length of the interview was 45–60 min. All interviews were audio recorded and transcribed verbatim. Identifying information was removed at the point of transcription and pseudonyms were used hereafter.

### Data analysis

Interpretive phenomenological analysis (IPA) is a useful methodological framework in qualitative psychology that draws upon the epistemological foundation of phenomenology, hermeneutics, and idiography. The primary goal of IPA is to investigate how individuals make sense of their experiences, assuming people are ‘self-interpreting beings’. The richness of the data produced by the semi-structured qualitative interview and the flexibility of interpretation in IPA was considered suitable for exploring individuals’ experiences, particularly complex and difficult ones [35]. Following steps outlined by Smith [34], transcripts were read and re-read multiple times, reflections and observations noted, notes were transformed into emergent themes, and clustered based on conceptual similarities. To improve validity of the themes which emerged from the transcripts, both KE and TWH reviewed the material. Illustrative quotes were selected to exemplify less self-explanatory themes and to substantiate the findings. Quotes from the transcripts have been translated from Norwegian to English and edited for legibility, with explanations added in square brackets if required and are identified by initials to protect anonymity.

## Results

As shown in Table 1, three main themes emerged from the material: (1) dealing with the focus upon physical appearance while battling a mental illness, (2) “project perfect”: feeling pressure to prove oneself, and (3) the importance of being seen and understood.

**Table 1:** Overview of main themes and subthemes

Themes and subthemes	Number of participants
1. Dealing with the focus upon *physical* appearance while battling a *mental* illness	
(a) You are not skinny enough to have an ED!	5 of 7
(b) Weight-based access to treatment	7 of 7 (clearly in 5)
2. Project perfect: Feeling pressure to prove oneself	7 of 7 (clearly in 5)
3. The importance of being seen and understood	
(a) The lack of congruence between the internal experience and the response from the external environment	7 of 7
(b) Having someone to fight for you	7 of 7 (clearly in 5)

## Dealing with the focus upon one’s physical appearance while battling a mental illness

This main theme revolved around the excessive, and untoward,  attention that others place upon one´s physical appearance and weight while psychological aspects of the illness are neglected. The first subtheme captures being overlooked or under-recognized by others, including friends, family, school or health professionals, etc. largely due to not being considered “thin enough” to have an ED. The second subtheme is specific to treatment settings, and captures the viewpoint that access to treatment, admission and discharge, is often reliant on the physical measure of body weight.

### Not skinny enough to have an ED

Several of the participants shared that friends and family never believed they struggled with an eating disorder. This negatively affected participants, resulting in feelings of humiliation, shock, confusion, shame and feeling invisible. One participant shared,

“I have friends from high school, that are still my friends today that [they] don’t believe I ever struggled with eating disorders. And that’s friends who are supposed to be close friends, and it hurts a lot. I was skinny when I was actively struggling with the eating disorder, but when I started to recover and reached normal weight…. yeah, they point blank don’t believe that I’ve ever had an ED because they didn’t see me as being skinny enough.” -Thea

Participants reported that these incidents caused self-doubt, and created a feeling of uncertainty over whether they actually were sick and deserved treatment. When one participant disclosed she had an eating disorder to a group of peers, she recalled,

“…It was very shameful, I felt, yeah, it was embarrassing to talk about it, and stuff like that. But, I thought, let’s see if I am brave enough to do this. And then afterwards, almost everyone came up to me surprised and said “God, if you have an eating disorder…I know people [with EDs], but they are super-skinny, and you are not that skinny!” -Hege

These incidences illustrate some of the effects external comments had on the participant, as well as revealed the tendency to be concerned with how one was perceived by the surroundings. Several participants reacted to the fact that people would comment on their appearance but not ask how they were doing emotionally, and several were bothered by comments on the physical aspects. Several participants also described troubling incidents following recovery after weight restoration, in which others made weight-based comments seemingly without knowledge of the seriousness of an eating disorder:

“What I found maybe most difficult about it [weight gain] was that my fellow students who didn’t know me very well, we were a pretty large class, right, and the ones that saw me every day and didn’t know that I had been very sick, suddenly I’d gained a lot, (…) I was a little bit ashamed of having gained that much weight because I was thinking that now, they don’t know… that I was life threateningly ill, sort of, so it is important that I gain weight.” -Marie

#### Weight-based threshold to access treatment

The need to become thinner, or “worse,” to access treatment, or before being taken seriously by health care professionals, was a major theme for all participants. When one participant recognized a spike in ED symptoms following a break-up, she reached out for professional help to avoid a relapse,

… “But they told me “no”, they sort of just blew me off and said they just needed to monitor this, but there’s nothing now that indicates that we need to take action because your weight is relatively stable, and your blood samples are fine”. - Stine

She further explained that her difficulties increased, she was eventually referred for treatment a second time, yet she was stunned at the intensity her illness needed to reach before she was taken seriously.

“After multiple rejections then, why, what’s the point then? Why should I fight for something that nobody cares to fight for with me? Like three months went by until the doctor realized that this [the relapse] was serious, but by then my weight had gone drastically down. And that still annoys me today…”

## Project “perfect”: under pressure to prove oneself

The second main theme that emerged from the data reflected the self-ascribed importance of being successful at the ED, to “prove” oneself to be “sick” enough, and for some, this had occurred within a context of perfectionistic and competitive traits. Several participants tied the onset of the illness to gaining control. The illness was described as an “area of expertise” and many participants were motivated to be “the best” at the ED. Being viewed as “not sick enough” dealt a blow to this endeavor, and in some instances, prompted additional weight loss and a deterioration in symptoms. One participant recalled a visit to a school nurse,

“There I experienced… that I wasn’t (…) sick enough. So, I remember that I, I had sessions with her once a week, or once a month, I don’t remember exactly, anyways I remember that I made myself a goal that I would be f******* sick enough for her to tell me that “now you need help”. - Tiril

One participant described her ability to restrict made her feel special and unique, especially as most people tried yet failed to lose weight:

“Eh, and I got sort of an uncomfortable relationship with my weight, and then it was the summer before I started the second year of high school and I thought, “I’m gonna lose weight. But there are so many that lose weight and then they gain it again, but that’s not me,” I told myself. “*I’m* going to make it”. - Thea

## The importance of being seen and heard

When looking at the material as a whole, the importance of being seen and understood was perhaps the most salient theme. Two subthemes emerged, the first of which captured experiences in dealing with an invalidating environment, where one’s external surroundings directly or indirectly contradicted or minimized the internal feelings, beliefs, and experiences of the individual. The second subtheme reflected the importance of having someone available who is not only understanding and supportive, but who remains steadfast and persistent in encouraging and enabling, treatment motivation and access.

### The lack of congruence between the internal experience of the individual and the response from the external environment

All of the participants described situations in which they felt misunderstood, or their symptoms were trivialized, leading to a sense of confusion and hurt. Failed attempts to acquire support and help left several participants feeling demotivated and uncertain.

“When I was admitted to the ER and was about to be discharged, I was told by a doctor there that I was supposed to just eat (…) three potatoes and two meatballs and then it will be OK. So just eat *that*, *then* everything will be fine, you’ll see… And this is at the ER (…). So, comments from health professionals that I’ve gotten are completely out of touch, where I don’t think they understand how sick you really are.” -Tiril

Most of the participants shared detailed accounts of aspects of their illness that were dramatic and scary, yet these experiences were often discounted or trivialized by others. This led to feelings of uncertainty and distrust, particularly towards the treatment system, and led to withdrawal and unwillingness to share additional information. One participant said this when describing her first meeting with her GP (general practitioner), after she disclosed that she had eating problems:

“She started off by asking me questions, and they were really weird ones, like, “are you gay?”, “have you (…) had a traumatizing childhood?”, and all those things I answered “no” to…Then when she asked me how I ate on a normal day. I explained how I had eaten in January, when things were going well. Now things were much worse. But by that point in the assessment, I didn’t dare to tell her….” - Maria

### Having someone to “fight” for you

In contrast, the participants ardently recalled occasions during which they had felt seen and heard, naming specific encounters and individuals who “fought for them.” One of the participants repeatedly mentioned her GP, and when asked what she would do if she felt at risk for a relapse, she said, without hesitation, that she would talk to her GP,

“…because he is sort of the one who saved my life. If I lost some weight or like, hadn’t managed what we had agreed on as my goals, then it was never like I was hopeless, but more like, “okay, we’ll have to try again and then we’ll have to see (…) where we went wrong and we’ll try and see if we can find another way that works better for you…” Lise.

Another participant recalled feeling supported and validated by the sheer determination of a treatment provider, which was turning point in her recovery by boosting her motivation and lessening her ambivalence to engage in treatment.

“… And then she said, “What did you just say? Oh my god, you can’t leave here before we do something about this. Have you ever gotten help? After I replied no, she immediately called someone and I got an appointment right then and there, and she followed me to my appointment. So, I kind of realized that I wanted the help, you know.” - Hege

One participant shared how she was first denied for local specialized treatment after being referred by her GP:

“He [the GP] said, “that’s not good enough, we can’t accept that. After that, I got an appointment quite fast, and I received the diagnosis of atypical anorexia.” - Lise.

On the other hand, some participants recalled failed opportunities to be understood and taken care of by loved ones. One participant mentioned that her parents commented on her losing weight but they did not take action, and although it was a *“good comment to get*” she left feeling unseen and unsupported:

“I’m left feeling very betrayed, sort of. Very betrayed, very, not to be seen, but I thought after that, that in fact your own parents didn’t do anything because they thought it would pass.” - Hege

Another participant remarked,

“Mom said lots of times, like, “oh, now your losing… just don’t lose control”, and I was like “no, no, no, it is just a couple of kilos”. But I didn’t have control whatsoever….it’s very much like… “Okay, we can see that there is something wrong with her, but we won´t ask her about it.” Tiril.

## Discussion

To our knowledge, this is the first study to explore the perspectives of individuals with a history of AN or AAN who have experienced being viewed as “not sick enough” by others at some point during their course of illness. Semi-structured interviews were administered face-to-face, and interpretive phenomological analysis was used to explore the in-depth experiences and perceived effects on prognosis, course, symptom development and motivation for recovery. Three main themes emerged from the interviews, (1) dealing with the focus upon one’s physical appearance while battling a mental illness, (2) “project perfect”: feeling pressure to prove oneself, and (3) the importance of being seen and understood.

The first main theme centered on the tendency of others to focus on physical measure of illness, such as weight, while psychological aspects of the illness were overlooked or under-recognized. All of the study participants had experienced at least one episode during the course of illness in which they perceived their symptoms were disregarded or trivialized by others—based on physical measures––as not serious enough to warrant, access, or continue treatment. Participants recalled feeling a variety of negative emotions in reaction to this experience, from annoyance and confusion, to despair, lack of trust, and an increased ambivalence to treatment and unwillingness to disclose information. A few participants directly linked trivialization of symptoms to an intentional intensification of restrictive eating behavior in an effort to “prove” their illness or to gain access to treatment. These findings are consistent with prior research detailing deleterious consequences in which individuals deliberately attempted to lose more weight to access treatment [28].

Relating to the second theme, one aspect which surfaced during the interviews was the desire to “be successful or good” at having an ED. In many ways, having an ED made the individual feel special and unique, and control over eating and weight provided a sense of mastery. Other studies have reported similar findings, that the ED provides the individual a feeling of being special, and this is generally described as one of the positive things connected to the illness [36]. In response to being perceived as “not sick enough,” several participants reported they needed to “prove” the seriousness of their illness. Other research has also highlighted the interplay between a competitive nature of eating disorders and a weight-based access to treatment, in which individuals feel successful and taken seriously at lower weights [27].

The final theme related to the importance of being “seen” and “heard”. This was an overarching theme and consistent with recent findings suggesting that the interaction of individuals with AN and their social environment, and specifically, the recognition of illness by family and peers, often functions as a facilitator in seeking timely help [12]. Several studies have confirmed that unconditional support from significant others, family, friends and health care providers is an important resource for an individual in recovery from EDs [37], and is related to lower levels of emotional distress and improved well-being generally [38]. Family support, and being believed when disclosing difficulties, has also proven important for seeking help and completing treatment [39, 40]. Some of the participants recalled feeling unseen or unsupported by family members who clearly recognized a problem yet didn’t take action or ask questions. Feeling supported by healthcare providers is also a particularly salient predictor of treatment engagement and satisfaction [41]. Several of the participants recalled the exact moment, or series of moments, during which healthcare providers advocated, sometimes relentlessly, for a referral or admission. Being “fought for,” despite privately harboring some level of ambivalence or uncertainty about the need for treatment, was identified by several participants as a turning point in motivation for recovery.

## Clinical implications

This study offers novel evidence that, from the perspective of service users, that relying upon BMI as a marker for the presence or severity of an eating disorder may have adverse consequences to well-being and treatment motivation, in addition to mischaracterization or misclassification of health status [42, 43]. As such, our findings support prior arguments that treatment must “move beyond skinniness” [44] by not solely focusing on weight restoration, and address psychological and psychosocial aspects “from an early stage” [27].

Another implication of this study is that a weight-based approach to treatment is insufficient across all stages of the illness and treatment, from prodromal to residual phases. Waiting until significant weight loss occurs counteracts efforts to intervene early, and as observed in the present study, leaves patients feeling frustrated, less motivated to engage in treatment, and unsupported. Our participants also shared experiences of being discharged based upon meeting a weight threshold, without sufficient regard to the level of ED psychopathology, or conversely, being denied readmission following a relapse regardless of spikes in ED-related behaviors and cognitions. Indeed, one participant recalled frustration during a relapse, as she was not offered treatment until after three months of rapid weight loss had ensued. A focus on weight restoration without accompanying improvements in psychological, cognitive, and psychosocial function, for instance, is reflected in high relapse rates for AN [45], and indeed, several have argued for a broader focus beyond weight to define recovery and determine health needs [46, 47].

Within this context, it is important to note that an extremely low body weight due to malnutrition or starvation indeed propels a cascade of medical sequelae with potentially detrimental effects [48]. Nevertheless, prior studies have shown that a lack of currently low weight does not preclude medical compromise [2, 49]. One study, for example, found that a 5% weight loss in combination with significant ED-related cognitive concerns (i.e.., undue influence of weight on self-evaluation and fears of weight gain) was a marker for a clinically meaningful restrictive eating disorder [50]. This also concurs with increasing evidence suggesting the importance of assessing relative weight status and weight history, not simply the current weight of the individual, when determining health status and treatment needs [51] [3].

## Study limitations and future directions

Limitations of this study are important to mention. This study used a purposive sampling strategy to explore individual range of perspectives and experiences, and did not aim to generalize findings. Participants were self-selected and recruited via social media on user organization websites, and had previously sought treatment, and may have differed markedly from cases in the general community. Few individuals in the community with a diagnosable eating disorder seek treatment, and individuals with atypical symptoms seek treatment less often than the ones with more stereotypical presentations [23]. The present sample included recovered individuals willing to share their retrospective experiences, and data may be subject to recall or memory biases. The reflections of persons with lived experiences are valuable, and provide insight spanning the course of illness, including recovery and relapses; however, interviewing a current clinical sample, however, might have yielded different results.

Participants were screened by phone for eligibility based upon self-reported diagnostic status, but no diagnostic interview was administered to confirm current or past medical or psychiatric status or weight history. Additional information regarding diagnostic history, along with demographic and illness-related variables such as length of illness and time since recovery would be useful for the contextualization of findings, and future quantitative studies are encouraged to examine these variables in relation to participants’ subjective experiences. In the current study, weight history, including current and past level of underweight, was not specifically related to the experiences of not feeling “sick enough”, although this would also be an interesting area of future research. Additionally, further relevant aspects, such as insight into the disorder (self-evaluation of being sick enough, readiness to change), or age of onset, would be similarly interesting to explore in future investigations. We opted to include individuals with either a past diagnosis of AN or atypical AN in an effort to allow for diagnostic progression and inconsistency between providers in diagnostic practices, and a stricter approach to the inclusion criteria may have yielded different results.

Although both genders were eligible for participation, no males were identified via the recruitment process. Future research is needed to explore the views and experiences of ethnically and culturally diverse groups, as well as males, across the lifespan whose illness may be overlooked or trivialized due to a non-stereotypical presentation. It would also be interesting to broaden the scope to examine other types of eating disorders, for example, binge eating disorder, bulimia nervosa, avoidant-restrictive food intake disorder. A review of studies investigating public and healthcare professionals’ knowledge and attitudes toward binge eating disorder, for instance, revealed limited public awareness that BED constitutes a diagnosable eating disorder [52]. Future research is also recommended to explore the experiences of carers. One study has found that carers of persons with mental illness experienced difficulties accessing services for their loved ones, being denied access due to their relative's illness being classified as “not in crisis” which had created feelings of frustration, distress and anxiety for families [53].

## Conclusions

In conclusion, findings may help provide a framework for further investigation of the experience of being “not sick enough” from a restrictive eating disorder. Qualitative research can facilitate an improved understanding of the perspectives and priorities of persons with lived experience and provide the groundwork for additional research. Increased awareness of diverse, non-stereotypical presentations of restrictive eating disorders is needed to challenge beliefs among healthcare providers and the public-at-large that body weight is the primary indicator of the presence or severity of illness. Results underscore the importance of social support and being “seen” and understood, and the potentially deleterious consequences of disclosing symptoms which are then trivialized or disregarded by others. Assertively advocating, or being “fought for,” by healthcare providers was viewed as especially powerful in motivating willingness to engage in treatment when battling a restrictive eating disorder.

## Data Availability

The dataset used and/or analyzed during the current study are available from the corresponding author on reasonable request.
